# Gallbladder cancer-associated fibroblasts promote vasculogenic mimicry formation and tumor growth in gallbladder cancer via upregulating the expression of NOX4, a poor prognosis factor, through IL-6-JAK-STAT3 signal pathway

**DOI:** 10.1186/s13046-020-01742-4

**Published:** 2020-11-05

**Authors:** Mu-Su Pan, Hui Wang, Kamar Hasan Ansari, Xin-Ping Li, Wei Sun, Yue-Zu Fan

**Affiliations:** 1 Department of Surgery, Tongji Hospital, Tongji University School of Medicine, Tongji University, Shanghai, 200065 P.R. China; 2 Department of Surgery, Shanghai Tenth People’s Hospital, Tongji University School of Medicine, Tongji University, Shanghai, 200072 P.R. China

**Keywords:** Gallbladder neoplasm, Gallbladder cancer-associated fibroblasts, Vasculogenic mimicry, NOX4, Signaling pathway, Prognosis

## Abstract

**Background:**

Cancer-associated fibroblasts (CAFs) and vasculogenic mimicry (VM) play important roles in the occurrence and development of tumors. However, the relationship between CAFs and VM formation, especially in gallbladder cancer (GBC) has not been clarified. In this study, we investigated whether gallbladder CAFs (GCAFs) can promote VM formation and tumor growth and explored the underlying molecular mechanism.

**Methods:**

A co-culture system of human GBC cells and fibroblasts or HUVECs was established. VM formation, proliferation, invasion, migration, tube formation assays, CD_31_-PAS double staining, optic/electron microscopy and tumor xenograft assay were used to detect VM formation and malignant phenotypes of 3-D co-culture matrices in vitro, as well as the VM formation and tumor growth of xenografts in vivo, respectively. Microarray analysis was used to analyze gene expression profile in GCAFs/NFs and VM (+)/VM (−) in vitro. QRT-PCR, western blotting, IHC and CIF were used to detected NOX4 expression in GCAFs/NFs, 3-D culture/co-culture matrices in vitro, the xenografts in vivo and human gallbladder tissue/stroma samples. The correlation between NOX4 expression and clinicopathological and prognostic factors of GBC patients was analyzed. And, the underlying molecular mechanism of GCAFs promoting VM formation and tumor growth in GBC was explored.

**Results:**

GCAFs promote VM formation and tumor growth in GBC; and the finding was confirmed by facts that GCAFs induced proliferation, invasion, migration and tube formation of GBC cells in vitro, and promoted VM formation and tumor growth of xenografts in vivo. NOX4 is highly expressed in GBC and its stroma, which is the key gene for VM formation, and is correlated with tumor aggression and survival of GBC patients. The GBC patients with high NOX4 expression in tumor cells and stroma have a poor prognosis. The underlying molecular mechanism may be related to the upregulation of NOX4 expression through paracrine IL-6 mediated IL-6/JAK/STAT3 signaling pathway.

**Conclusions:**

GCAFs promote VM formation and tumor growth in GBC via upregulating NOX4 expression through the activation of IL-6-JAK-STAT3 signal pathway. NOX4, as a VM-related gene in GBC, is overexpressed in GBC cells and GCAFs, which is related to aggression and unfavorable prognosis of GBC patients.

## Introduction

Gallbladder cancer (GBC) is a highly malignant primary tumor of the biliary tract. Due to the lack of specific clinical manifestations and effective monitoring indicators, the early diagnosis rate of GBC is low. At the same time, the disease also has the characteristics of high invasion and metastasis, insensitivity to chemo-radiotherapy, resulting in disappointing surgical and drug treatment results and poor prognosis; according to relevant reports, its 5-year survival rate is only about 5% [1–3]. Therefore, it is of great significance to understand the pathogenesis of GBCs and explore the effective prognosis indicators and drug targets, so as to improve the diagnosis, treatment and prognosis of GBC patients.

It is generally believed that the occurrence and development of tumor is not only related to tumor itself, but also depends on the complex interaction between malignant cells and their microenvironment. Tumor microenvironment (TME), also known as tumor stroma, is composed of extracellular matrix (ECM) and a variety of stromal cells [4, 5]. Cancer-associated fibroblasts (CAFs) are the most important stromal cells in TME, which play an important role in tumor growth and development, and participate in many biological processes such as tumor proliferation, invasion and metastasis, angiogenesis [6–12], and are related to poor prognosis [13–15]. In these biological processes, angiogenesis and effective blood supply are the basic conditions for tumor growth and metastasis [16, 17]. In recent years, studies have found a novel tumor blood supply pattern, called vasculogenic mimicry (VM), which occurs in certain highly aggressive malignancies, and is closely related to poor clinical results and poor prognosis [18–21]. It has become one of the potential targets for anticancer therapy [22–25]. We previously reported that VM exists in GBC and is associated with the patient’s poor prognosis. The mechanism of VM formation in GBC may be closely related to the activation of PI3K/MMPs/Ln-5γ2 and/or EphA2/FAK/Paxillin signaling pathways [26–29]. Recently, it was reported that CAFs can promote VM formation in some malignant tumors, such as hepatocellular cancer (HCC) [30] and gastric cancer [31, 32]. However, so far, the mechanism of VM formation is not clear, and the relationship between gallbladder cancer-associated fibroblasts (GCAFs) and VM formation in GBC has not been reported.

NOX4 (nicotinamide adenine dinucleotide phosphate oxidase 4, NADPH oxidase 4) is a number of NADPH oxidase (NOX) family [33, 34]. NOX family plays vital roles in signaling transduction, cell growth, apoptosis, differentiation and tumor development. Long-term oxidation/reduction imbalance is considered to be one of the important factors leading to carcinogenesis. Oxidative stress induces the development of cancer due to excessive production of reactive oxygen species (ROS) by members of the NOX family [35–38]. It was reported that NOX4 is overexpressed in many malignancies and participates in malignant processes such as proliferation and metastasis [39–41]; NOX4 can also promote tumor angiogenesis by regulating the production of vascular endothelial growth factor, which further results in poor prognosis [42].

In this study, we aimed to study the relationship between GCAFs and VM formation and tumor growth in GBC, and to explore the related molecular mechanism. At the same time, we also studied the clinical value of overexpression of NOX4, which is a key gene for VM formation in GBC.

## Materials and methods

### Cell lines and cultures

Three established human GBC cell lines, GBC-SD (Shanghai Cell Biology Research Institute of Chinese Academy of Sciences, CAS, China), SGC-996 (Gift from Professor Yao-Qin Yang, Institute of tumor cytology, Tongji University School of Medicine), OCUG-1 (Gift from Professor Ying-Bin Liu, Shanghai Xinhua Hospital), and TJ-GBC2 (A novel GBC cell line, constructed in our laboratory [43]) were used in this study. These cells were propagated in Dulbecco’s modified Eadles medium (DMEM, Gibco, USA) supplemented with 10% fetal bovine serum (FBS, Ausbian, Australia) and 0.1% gentamicin sulfate (Gemini Bioproducts, Calabasas, Calif). The established human umbilical vein endothelial cell line (HUVEC, gift from Department of Pathophysiology, Shanghai Medical College, Fudan University) was cultured in endothelial cell medium (ECM, Sciencell, USA) with 10% FBS. Human GCAFs and normal fibroblasts (NFs) were isolated from the clinical specimens of human GBC tissues and adjacent normal tissues, and identified by the detection of the stromal markers α-smooth muscle actin (α-SMA) and fibroblast activation protein (FAP) using immunohistochemistry (IHC), co-immunofluorescence (CIF) and western blotting. The established GCAFs and NFs (the cells used in this experiment were between the 4th and 9th generation) were incubated in DMEM/F-12 medium (Gibco; USA) supplemented with 10% FBS. Co-cultures of GBC cells and GCAFs or NFs were performed as previously described [13]. All of the cells were maintained in a carbon dioxide (CO_2_) incubator (SANYO MCO-175, Japan) at 37 °C with a 5% CO_2_ atmosphere.

### VM formation assay in vitro

The Transwell chamber (aperture 0.4 μm, diameter 6.5 mm) was used to establish a co-culture system of human GBC cells and fibroblasts. Human GBC cell lines (GBC-SD, SGC-996 and OCUG-1) and fibroblasts (GCAFs or NFs) were used to prepare cell suspensions (GBC cells, 4 × 10^4^·ml^− 1^; fibroblasts, 2 × 10^4^·ml^− 1^). Matrigel and rat-tail type I collagen three-dimensional (3-D) matrices (ABI, USA) were prepared as described previously [20], coated on the bottom of the lower chamber respectively for comparative test. Cells were divided into GBC cell (GBC-SD), GBC cell (GBC-SD) + NFs and GBC cell (GBC-SD) + GCAFs groups. In the analysis of VM related gene expression profile, cells were divided into GBC cell (GBC-SD, SGC-996 or OCUG-1) group and GBC cell (GBC-SD, SGC-996 or OCUG-1) + GCAFs co-culture group. 50 μl GBC cell suspensions (5 × 104·ml^− 1^) were injected into the lower chamber containing different gels separately and incubated for 2 h, then DMEM/F12 medium containing 100 U·ml^− 1^ penicillin and streptomycin (containing 0.2% FBS) was added. The same amount of (200 μl) fibroblast suspensions (2 × 10^4^/ml) or serum-free medium were added to the upper chamber. The culture medium was changed every 2–3 days. The formation of VM in 3-D matrices from GBC cells or co-cultures was performed by hematoxylin and eosin (H&E) staining and immunohistochemical periodic acid-Schiff (PAS) staining (without hematoxylin counterstain) as described previously [20, 23, 29], and was observed under an inverted optical microscope (Caikang XDS-100) every day, and the number of VM formation was recorded in 5 visual fields at 200 magnifications.

### Assays of malignant phenotypes of GBCs triggered GCAFs in vitro

To verify that GCAFs promote VM formation of GBCs, in following experiments we detected malignant phenotypes including proliferation, invasion, migration and tube formation of GBC cells triggered by GCAFs in vitro.

Cell proliferation was assessed using CCK-8 method. Cells were divided into GBC cell (GBC-SD, SGC-996 or TJ-GBC2) group, GBC cell (GBC-SD, SGC-996 or TJ-GBC2) + NFs co-culture group and GBC cell (GBC-SD, SGC-996 or TJ-GBC2) + GCAFs co-culture group. A co-culture model of GBC cells and fibroblasts (GCAFs or NFs) was established by Transwell chamber (Corning, USA) with aperture of 0.4 μm and diameter of 6.5 mm. Lower chambers were inoculated with 700 μl GBC cell suspensions (4 × 10^4^/ml), upper chambers inoculated with 200 μl fibroblasts (GCAFs or NFs) suspensions (2 × 10^4^·ml^− 1^) or equal volume serum-free medium. After 24 h, 36 h and 48 h of culture, CCK-8 solution (10 μl/well) was added, and the culture was continued for 1 h. The optical density (OD) value of each well was measured by enzyme-labeled instrument (Elx800UV, BIO-TEK, USA) at 450 nm wavelength. All experiments were performed in triplicate.

Cell invasion was assessed using the Transwell chambers with aperture of 8 μm and diameter 6.5 mm (Corning, USA). Matrigel (200 μl/well, BD, USA) was coated on the bottom of the upper chamber to simulate the basement membrane and extracellular matrix. Cells were grouped as above. GBC-SD, SGC-996 and TJ-GBC2 cell suspensions (1 × 10^5^·ml^− 1^; 200 μl) were respectively inoculated on the gel in the upper chambers. Lower chambers were inoculated with 700 μl NFs, GCAFs (5 × 10^4^·ml^− 1^) or serum-free medium. After 24 h of culture, the cells invaded through the basement membrane were stained with Giemsa (Beyotime, China) and counted under an inverted optical microscope (Caikang XDS-100, Shanghai, China). All experiments were performed in triplicate.

Cell migration was assessed with a wound healing assay. Cells were grouped as above. 100 μl GBC cell suspensions (5 × 10^4^ cells/well) or equal volume co-culture cells (50 μl GBC cell suspensions, 50 μl NFs or GCAFs; 5 × 10^4^·ml^− 1^) containing NFs, GCAFs or serum-free medium were inoculated into 96-well wounding plate (Coster, USA) with culture medium and cultured in a single layer for 24 h until 90% cells fused. Then, a scratch tester was used to scratch a wound at the central bottom of 96-well plate. Cell migration areas were scanned and analyzed at 0 h, 8 h and 24 h using a Cellomocs (Thermo Fisher Scientific, USA), and observed under an inverted optical microscope (Caikang XDS-100) at 200 magnifications. Cell migration area (pixel area) = (S3 + S4) - (S1 + S2). All experiments were performed in triplicate.

Tube formation was assessed with the model of interaction between GBC cells, GCAFs, NFs and HUVECs established by using Transwell chamber (aperture 0.4 μm, diameter 6.5 mm). The bottom of 24-well plate was covered with Matrigel (200 μl/well) to provide 3-D growth space for HUVECs. Cell suspensions (HUVEC 1 × 10^4^·ml^− 1^; NFs or GCAFs 3 × 10^4^·ml^− 1^; GBC-SD 2 × 10^4^·ml^− 1^) were made by adding serum-free DMEM/F12 or ECM (HUVEC only). Cells were divided into HUVECs, HUVECs+GBC-SD, HUVECs+GBC-SD + NFs and HUVECs+GBC-SD + GCAFs groups, i.e. lower chambers were inoculated with 200 μl HUVECs; the upper chamber was respectively added with 200 μl serum-free medium, GBC-SD cell suspensions, GBC-SD + NFs cell suspensions or GBC-SD + GCAFs cell suspensions. During 48 h of cell culture, the lumen formation was observed dynamically under an inverted optical microscope (Caikang XDS-100), and the number of the lumen formed by HUVECs was counted. All experiments were performed in triplicate.

### Tumor Xenograft assay in vivo

The xenograft experiments were performed in accordance with the official recommendations of Chinese Community Guidelines, and were approval from Research Ethical Review Broad in Tongji University (Shanghai, China). BALB/C nu/nu mice (equal numbers of male and female mice, 4-week old, about 20 g) were purchased from Shanghai Laboratory Animal Center, Chinese Academy of Sciences, and housed under specific pathogen-free (SPF) conditions. The mice were randomly divided into GBC-SD group and GBC-SD + GCAFs group, 10 mice in each group. 0.2 ml serum-free medium containing GBC-SD or GBC-SD + GCAFs co-culture cell suspensions (5.0 × 10^6^·ml^− 1^) were respectively injected subcutaneously into the right axilback of the nude mice. Tumor xenograft size i.e. the maximum diameter (a) and minimum diameter (b) was measured with calipers twice a week. The tumor volume was calculated by the following formula: V (cm^3^) = Πab2/6. After 7 weeks, mice were sacrificed and xenograft specimens were used for western blotting, or were paraffin-embedded, deparaffinized, hydrated and were then used for immunohistochemistry (IHC) staining and Co-immunofluorescence (CIF) staining, respectively.

### VM formation assay of tumor xenografts in vivo

VM formation assay of xenograft sections in vivo was conducted by H&E staining, CD_31_-PAS double staining and transmission electron microscopy (TEM) as described previously [20, 23, 29]. Histomorphologic appearance and VM characteristic of the tumor xenografts in vivo were observed under an inverted optical microscope (Caikang XDS-100) and a JEOL-1230 TEM (Japanese Electronics, Japan).

### Patients and clinical specimens

From 2007 to 2011, 85 patients with GBC, 10 patients with gallbladder precancerous lesion or benign lesion were recruited from Tongji Hospital, Tongji University (Shanghai, China). This study was conducted in accordance with the official recommendations of ethical standards, the Declaration of Helsinki and the Chinese Community Guidelines, and was approved by the Ethics Committee and the Institutional Review Board of Tongji Hospital. A written informed consent was obtained from each patient. A total of 115 gallbladder tissue specimens including 105 paraffin-embedded specimens (85 GBC, 10 gallbladder precancerous or benign lesion specimens) and 10 fresh GBC specimens confirmed by operation and histopathology were used in this study. All GBC patients had not received chemotherapy or radiotherapy before surgery. Curative resection (R0 resection) was defined as no residual tumor status, whereas microscopic (R1 resection) and macroscopic residual tumor (R2 resection) was defined as non-curative resection. To reduce effects directly related to surgery, patients who died within 1 month after surgical resection were not included. Two independent pathologists who blinded to the patients’ clinical status verified diagnoses of these GBC samples. According to WHO criteria and the Nevin stage system, detailed clinicopathological and follow-up data were collected from the patient’s medical records and completed by a telephone survey, routine visit record and address. Clinical outcome was followed from the date of surgery to the date of death or until the end of September 30, 2011. Cases lost during follow-up were regarded as censored data for the survival analysis. The median follow-up period for all patients was 18.6 (range, 1–60) months. The 5-year overall survival (OS) rate was 11.8% (10/85). Demographic and clinicopathological data are summarized in Table 1.

**Table 1:** Correlation between NOX4 expression in tumor cells and clinicopathological parameters in patients with gallbladder cancer

Variable	NOX4 expression [n (%)]		***x***^**2**^ value (***P***)	Spearman rank correlation, r (***P***)
	Low	High		
**Age** (y)				
> 65	13 (40.6)	19 (59.4)	0.127 (0.649)	0.013 (0.834)
≤ 65	23 (43.3)	30 (56.7)		
**Gender**				
Male	26 (52)	24 (48)	1.153 (0.361)	0.244 (0.120)
Female	12 (34.2)	23 (65.8)		
**Tumor location**				
Bottom	20 (47.6)	22 (52.4)	0.689 (0.388)	0.105 (0.505)
Neck and other	15 (34.9)	28 (65.1)		
**Tumor size** (**cm**)				
> 3	19 (42.2)	26 (57.8)	0.734 (0.289)	0.125 (0.434)
≤ 3	19 (47.5)	21 (52.5)		
**Histological types**				
Adenocarcinoma	29 (35.8)	52 (64.2)	0.093 (0.672)	0.272 (0.345)
Other ^a^	1 (25)	3 (75)		
**Differentiation degree**				
High	7 (63.6)	4 (36.4)	7.676 (0.018) ^b^	0.422 (0.004) ^b^
Moderate	16 (47)	18 (53)		
Poor	8 (20)	32 (80)		
**Liver metastases**				
(+)	15 (33.3)	30 (66.7)	4.715 (0.030) ^b^	0.334 (0.032) ^b^
(−)	21 (52.5)	19 (47.5)		
**Vascular invasion**				
(+)	13 (27)	35 (73)	5.904 (0.025) ^b^	0.352 (0.028) ^b^
(−)	15 (40.5)	22 (59.5)		
**Lymph node metastasis**				
(+)	15 (33.3)	30 (66.7)	1.656 (0.198)	0.053 (0.635)
(−)	19 (47.5)	21 (52.5)		
**Nevin staging**				
III, IV, V	21 (32.8)	43 (67.2)	6.125 (0.019) ^b^	0.382 (0.025) ^b^
I, II	10 (47.6)	11 (52.4)		
**Resection method**				
R1, R2	14 (32.5)	29 (67.5)	1.660 (0.202)	0.055 (0.644)
R0	20(47.6)	22(52.4)		
**VM**				
(+)	18 (81.8)	4 (18.2)	6.625 (0.017) ^b^	0.321 (0.016) ^b^
(−)	12 (19.1)	51 (80.9)		

^a^squamous cell carcinoma, adenosquamous carcinoma; ^b^*P* < 0.05: statistically significant

### Affymetrix chip analysis on the gene expression profile for GCAFs/NFs and VM (+)/ VM (−) in vitro

Affymetrix GeneChip Human 1.0ST array (Affymetrix, USA) was used to analyze the gene expression profile in GCAFs/NFs in vitro. Briefly, after extracting total RNA in triplicate from GCAFs/NFs and testing the quality, 130 μl of the IVT Master Mix was added into to 130 μl of double-stranded cRNA using a GeneChip 3’IVT PLUS Kit (Affymetrix, USA) to perform RNA RT and in vitro transcription (IVT) of cRNA. Then the newly generated cRNA was synthesized, purified and labeled. Finally, after hybridizing and cleaning with a GeneChip Hybridization Wash and Stain kit (Affymetrix, USA), the Genechip Array scanner 3000 (Affymetrix, USA) was used to scan the assays to find out the differentially expressed genes between GCAFs and NFs. Array data were normalized by log scale robust multi-array analysis and analyzed by R-Project software. The gene expression was considered significant if the fold change (FC) value was > 1.5 or < 0.67, and *P* < 0.05. Gene Ontology (GO) analysis was used for functional enrichment analysis, and gene set enrichment analysis and Fisher exact analysis were used to perform statistical analysis of GO. In order to explore the differences of gene expression profile between GCAFs and NFs, potentially relevant up- or down-regulated genes involved in biological processes were selected for verification.

Affymetrix Human lncRNA array (Affymetrix, USA) was used to analyze the expression profile of VM related genes in VM (+) and VM (−) groups in vitro. Transcriptome library construction, transcriptome assembly and annotation protocols were provided by Shanghai Oe Biotech Co., Ltd., China. The Pearson correlation between its expression value and each mRNAs expression value was calculated for each lncRNA. For function prediction of lncRNAs, the co-expressed mRNAs for each differentiated lncRNA were calculated and then a functional enrichment analysis of this set of co-expressed mRNAs was carried out. The enriched functional terms were used as the predicted functional term of given lncRNA. The co-expressed mRNAs of lncRNAs were identified by calculating Pearson correlation with correlation *P*-value < 0.05. Then the hypergeometric cumulative distribution function was used to calculate the enrichment of functional terms in annotation of co-expressed mRNAs. The cis-regulation regions were identified by the following procedures. For each lncRNAs, we identified the mRNAs as “cis-regulated mRNAs” when: (1) the mRNAs loci are within 300 k windows up- and down-stream of the given lncRNA, (2) the Pearson correlation of lncRNA-mRNA expression is significant (*P*-value of correlation ≦0.05).

### Immunohistochemistry (IHC) and enzyme-linked immunosorbent assay (ELISA) in vitro and in vivo

IHC was used to detect the expression of NOX4 protein in sections from the 3-D co-culture samples in vitro, nude mice xenografts in vivo and human gallbladder tissues or stroma. After deparaffinizating and inactivating endogenous peroxides, the sections (4 μm thick) were pretreated with bovine serum albumin V working solution (Beijing Solarbio Science &Technology Co., China), then incubated with primary anti-rabbit NOX4 (Sigma, USA), secondary anti-rabbit IgG (Maixin, China) and 3, 3-diaminobenzidine (DAB) solution, and stained with hematoxylin according to the manufacturer’s instructions. Phosphate buffer saline (PBS; Thermo Fisher Scientific, USA) was used to replace the primary antibody for negative control. The expression of NOX4 was observed under an optical microscope (Olympus, Japan). In order to score the stains, five random fields of each section were observed or more than 500 cells counted per slide. In addition, the expression of NOX4 in different human gallbladder tissue/stroma was evaluated by a semi-quantitative system with the staining index (SI). The SI scoring criteria are as follows: (positive cell percentage score) (staining intensity score). The positive cell percentage was scored from 0 to 4 as follows: 0 (no positive cells), 1 (1–25%), 2 (26–50%), 3 (51–75%) and 4 (76–100%). The staining intensity was scored from 0 to 3 as follows: 0 (negative), 1 (weak), 2 (moderate), and 3 (strong). 3 of SI score were used to distinguish between low (IS ≤3) and high (IS > 3) protein expression.

The expression of interleukin-6 (IL-6) protein product in supernatant from the 3-D co-culture samples in vitro or nude mice xenografts in vivo was determined by ELISA using the Human IL-6 ELISA Kit (Abcam, UK) according to the manufacturer’s protocol. All samples were analyzed in triplicate.

### Co-immunofluorescence (CIF) staining in vivo

CIF staining with stromal markers such as α-SMA and FSP-1 was used to confirm the expression of NOX4 in GBC stroma. The above IHC samples were permeabilized in PBS containing 10% methanol for 30 min, washed in PBS, and sealed with PBS containing 3% FBS for 1 h. The M.O.M kit (Vector Laboratories, Inc., USA) was used to block Mouse IgG according to the manufacturer’s instruction. For CIF staining of NOX4 and α-SMA or FSP1, the sections were respectively incubated with rabbit anti-NOX4 (1:500; GeneTex, USA) and mouse anti-α-SMA (1:200; Abcam, UK) or mouse anti-FSP-1 (1:100; Abcam, UK) at 4 °C overnight. Then, the sections were incubated with corresponding secondary antibodies, goat anti-rabbit IgG (1:1000; Abcam, UK) for detecting NOX4 expression, goat anti-mouse IgG (1:200; Abcam) for α-SMA, goat anti-rabbit IgG (1:200; Abcam) for FSP1. Finally, the sections were washed in PBS and stained with diamidine phenylindole (DAPI) for 5 min, and observed under an immunofluorescence microscope.

### qRT-PCR in vitro

Quantitative reverse transcription-polymerase chain reaction (qRT-PCR) was used to verify the different expression of NOX4 at mRNA level in GCAFs/NFs and the expression of JAK1, JAK2 and STAT3 mRNAs of the IL-6/JAK/STAT3 signal pathway genes in GCAFs-triggered VM formation of GBC cells in vitro. Total RNA was extracted from cultured or co-cultured cells by Trizol reagent (Thermo Fisher Scientific, USA). GAPDH primers were used as the control for PCR amplification. The gene-specific primer sequences of NOX4, IL-6/JAK/STAT3 signal pathway genes and housekeeping gene GAPDH were as follows: NOX4, Forward: 5′-GTG TCT AAG CAG AGC CTC AGC ATC-3′, Reverse: 5′-CGG AGG TAA GCC AAG AGT GTT CG-3′; IL-6, Forward: 5′-GTG GAC CTG ACC TGC CGT CTAG-3′, Reverse: 5′-GAG TGG GTG TCG CTG TTG AAG TC-3′; JAK1, Forward: 5′-CAT CGT GAT CTT GCT GCT CAG-3′, Reverse: 5′-ACT CCI TGA TGC ACC ATA CGT C-3′; JAK2, Forward: 5′-TCC TCA GAA CGT TGA TGG CAG-3′, Reverse: 5′-ATT GCT TTC CTT TTT CAC AAG AT-3′; STAT3, Forward: 5′-GAG AAG GAC ATC AGC GGT AAG-3′, Reverse: 5′-AGT GGA GAC ACC AGG ATA TTG-3′; GAPDH, Forward: 5′-CTC CTC CTG TTC GAC AGT CA-3′, Reverse: 5′-GCT CCG CCC AGA TAC CATT-3′. The PCR amplification reaction was as follows: 94 °C for 3 min, followed by 40 cycles of 95 °C for 15 s, 60 °C for 30s, 72 °C for 30s, and 82–86 °C (fluorescence collection) for 5–10s, and finally 72–99 °C for 5 min. The PCR product (10 μl) was placed on 15 g·l^− 1^ agarose gel and observed by ethidium bromide (Cusabio Biotech, China) staining with ABI-Prism 7300 SDS software (Bio-Rad Laboratories, USA).

### Western blotting in vitro and in vivo

Western blotting was used to verify the expression of NOX4 protein and the expression of IL-6, JAK1, JAK2 or STAT3 protein in the 3-D culture/co-culture samples in vitro and nude mice xenografts in vivo. The total protein was isolated from the 3-D culture/co-culture samples and nude mice xenografts with RIPA (radioimmunoprecipitation assay) Lysis Buffer (SBJBIO, China), and the concentration was detected using BCA protein assay kit (Kangchen BioTech, China). Then, an aliquot of 20 μg proteins was subjected to 10% SDS-PAGE (sodium dodecyl sulfate-polyacrylamide gel electrophoresis) under reducing conditions, and proteins were transferred to a PVDF (polyvinylidene difloride) membrane (Millipore, USA). The membrane was incubated with the primary rabbit anti-NOX4 antibody (1:3000; Abcam, UK), anti-IL-6 antibody (1:3000; KangChen Biotech, China, the same below), anti-JAK1 antibody (1:2000), anti-JAK2 antibody (1:2000), anti-STAT3 antibody (1:1500) and mouse anti-human β-actin antibody (1:1000), and then the appropriate dose of horseradish peroxidase labeled anti-mouse/rabbit secondary antibody (1:1000; Kangchen BioTech) was added for further incubation. The target proteins were displayed by an enhanced chemiluminescent (ECL) kit (Kangchen BioTech), and imaged on a chemiluminescence imager. The gray value and gray coefficient ratio of every protein were analyzed and calculated with Image J analysis software (National Institutes of Health).

### Statistical analysis

All data were expressed as mean ± SD (standard deviation), and statistically analyzed by SPSS 22.0 software (IBM, USA). The SI was analyzed using Kruskall-Wallis test with Dunn’s post-hoc comparison. Student *t*-test was used in independent sample analysis. The χ^2^ test was used to analyze the relationship between NOX4 expression and GBC patients’ clinicopathologic parameters. Bivariate correlations between two independent variables were analyzed by calculating the Spearman’s correlation coefficients. The survival analysis was calculated by Kaplan-Meier method and compared by logrank test. The Cox’s regression model was used for univariate and multivariate analysis of prognostic factors. *P* < 0.05 was considered to be statistically significant.

## Results

### GCAFs promote VM formation of GBC cells in vitro

**Fig. 1:**
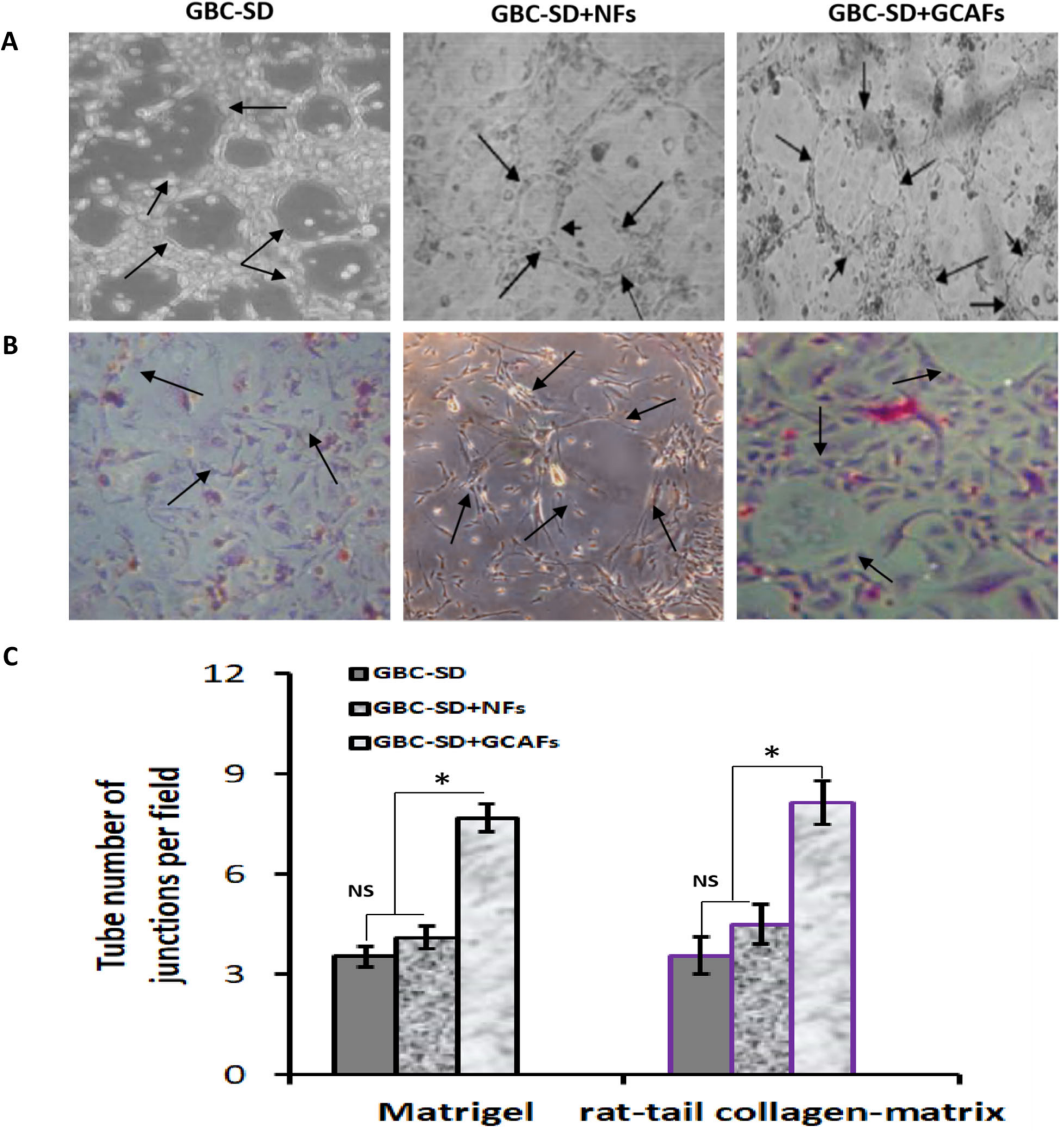
GCAFs promote VM formation of GBC-SD cells in vitro (Phase contrast microscopy on 3-D cultures, × 200; **A:** Matrigel, **B:** rat-tail collagen-matrix). The tube number of vasculogenic-like networks in GBC-SD + GCAFs groups was significantly more than that of GBC-SD and GBC-SD + NFs groups (all *P < 0.05); furthermore, PAS positive, cherry-red materials found in granules and patches in the cytoplasm of GBC-SD cells appeared around the signal cell or cell clusters

A 3-D co-culture system of GBC-SD and GCAFs/NFs was established to investigate the effect of GCAFs on the VM formation of GBC. As showed in Fig. 1, GBC-SD cells and GBC-SD + NFs groups formed vasculogenic-like networks when cultured on Matrigel and rat-tail collagen-matrix for 2 days, and had VM formation for 14 days; but GBC-SD + GCAFs group formed patterned vasculogenic-like networks for only 19 h, had VM formation for only 1 week; and the tube number of vasculogenic-like networks in GBC-SD + GCAFs was significantly more than that of alone GBC-SD cells and GBC-SD + NFs (*P* < 0.05). Furthermore, PAS positive, cherry-red materials found in granules and patches in the cytoplasm of GBC-SD cells appeared around the signal cell or cell clusters. These results showed that GCAFs can promote VM formation of GBC-SD cells in vitro.

### GCAFs promote the proliferation, invasion, migration and tube formation of GBC cells in vitro

**Fig. 2:**
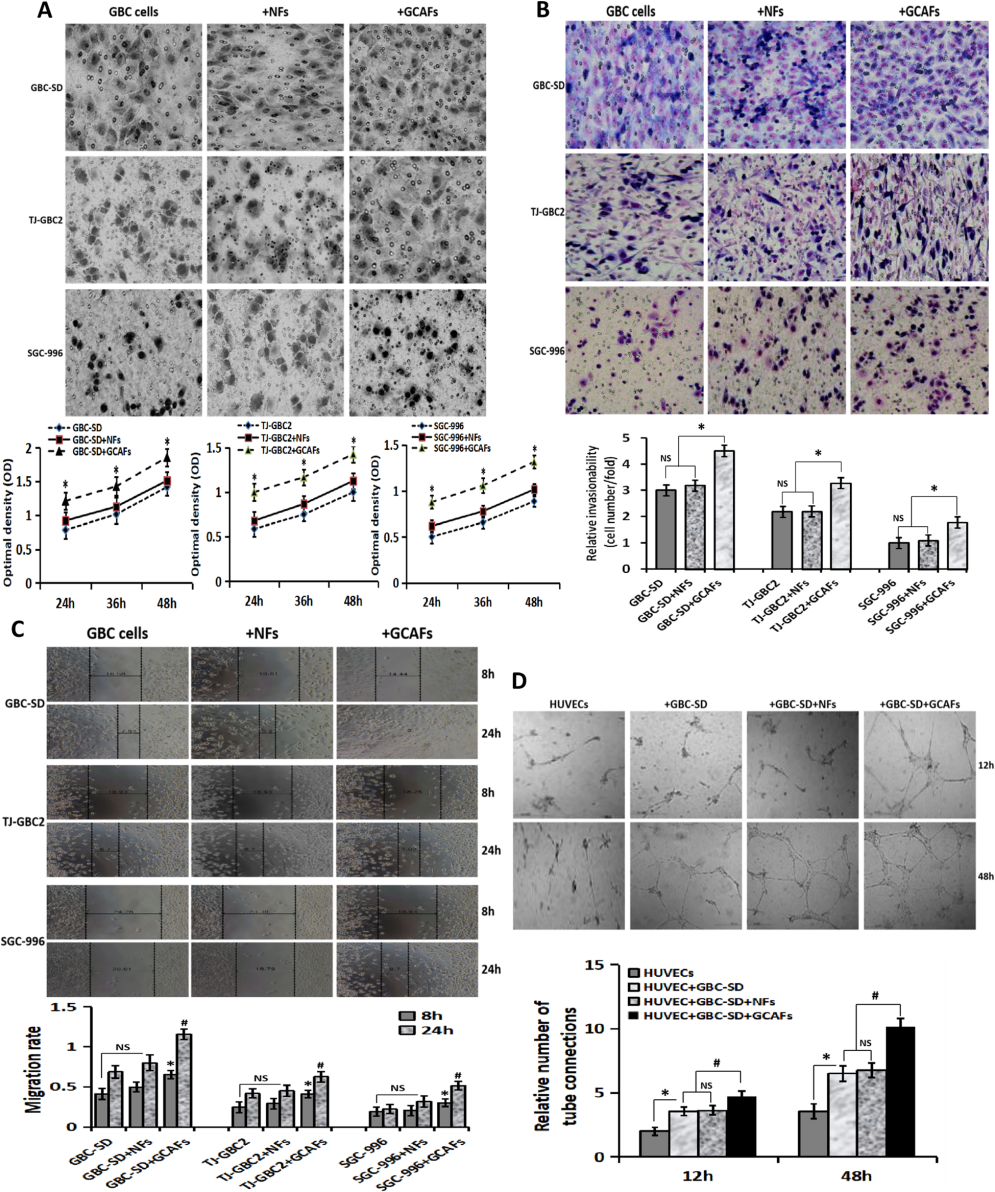
GCAFs promote the malignant phenotypes of GBC cells/HUVECs. **A**, Proliferation assay. Compared with NFs, GCAFs significantly promoted the proliferation of GBC cells at 24 h, 36 h and 48 h (vs. GBC cells group and GBC cells+NFs group, all *P < 0.05). **B**, Transwell invasion assay (Giemsa stain, × 200). The number (relative invasion ability) of cells that invaded through the basement membrane in GBC cell+GCAFs group was significantly more than that of GBC cell group and GBC cell+NFs group (all *P < 0.05). **C**, Wound healing assay. The cell migration rate of GBC cell+GCAFs group was significantly stronger than that of GBC cell group and GBC cell+NFs group at 8 h and 24 h (all ^#^*P* < 0.05). **D**, Tube formation assay. At 12 h: HUVEC group (−); HUVEC+GBC-SD and HUVEC+GBC-SD + NFs (+), (vs. HUVEC, all *P < 0.05); HUVEC+GBC-SD + GCAFs (+), (vs. HUVEC+ GBC-SD or HUVEC+GBC-SD + NFs, all ^#^*P* < 0.05). At 48 h: obvious tubular formation was observed in all the four groups, the number of tube formed in HUVEC+GBC-SD + GCAFs group was significantly more than that in the other three groups (all ^#^*P* < 0.01), but no statistical difference was observed between HUVEC+GBC-SD group and HUVEC+GBC-SD + NFs group (*P* > 0.05)

To verify that GCAFs promote VM formation of GBC cells, we detected malignant phenotypes including proliferation, invasion, migration and tube formation of GBC cells triggered by GCAFs in vitro using a co-culture model of GBC cells (GBC-SD, SGC-996, TJ-GBC2) and fibroblasts (GCAFs, NFs). As shown in Fig. 2, the proliferation ability of GBC cell+GCAFs groups were significantly enhanced compared to alone GBC cell or GBC cell+NFs groups (all *P* < 0.05; Fig. 2A); the number (relative invasive ability) of cells that invaded the basement membrane in GBC cell+GCAFs groups was significantly more than that of alone GBC cell or GBC cell+NFs groups (all *P* < 0.05; Fig. 2B); the cell migration rate in GBC cell+GCAFs co-culture groups was significantly higher than that of alone GBC cell groups or GBC cell+NFs co-culture groups (all *P* < 0.05; Fig. 2C). Furthermore, tube formation assay showed that tube structure was observed in HUVEC+GBC-SD + GCAFs groups at 12 h; no obvious tube structure was seen in HUVECs or HUVEC+GBC-SD + NFs cultured in Matrigel for 12 h, and the formation of tube structure cannot be observed until about 18 h. At 48 h, obvious tube formation was observed in HUVEC, HUVEC+GBC-SD, HUVEC+GBC-SD + NFs and HUVEC+GBC-SD + GCAFs groups; but among them, the number of the lumen formed in HUVEC+GBC-SD + GCAFs group was significantly more than that of the other three groups (all *P* < 0.01; Fig. 2D). Taken together, GCAFs can promote the proliferation, invasion, migration and tube formation of GBC cells/HUVECs, and then promote VM formation.

### GCAFs promote VM formation and tumor growth of GBC xenografts in vivo

**Fig. 3:**
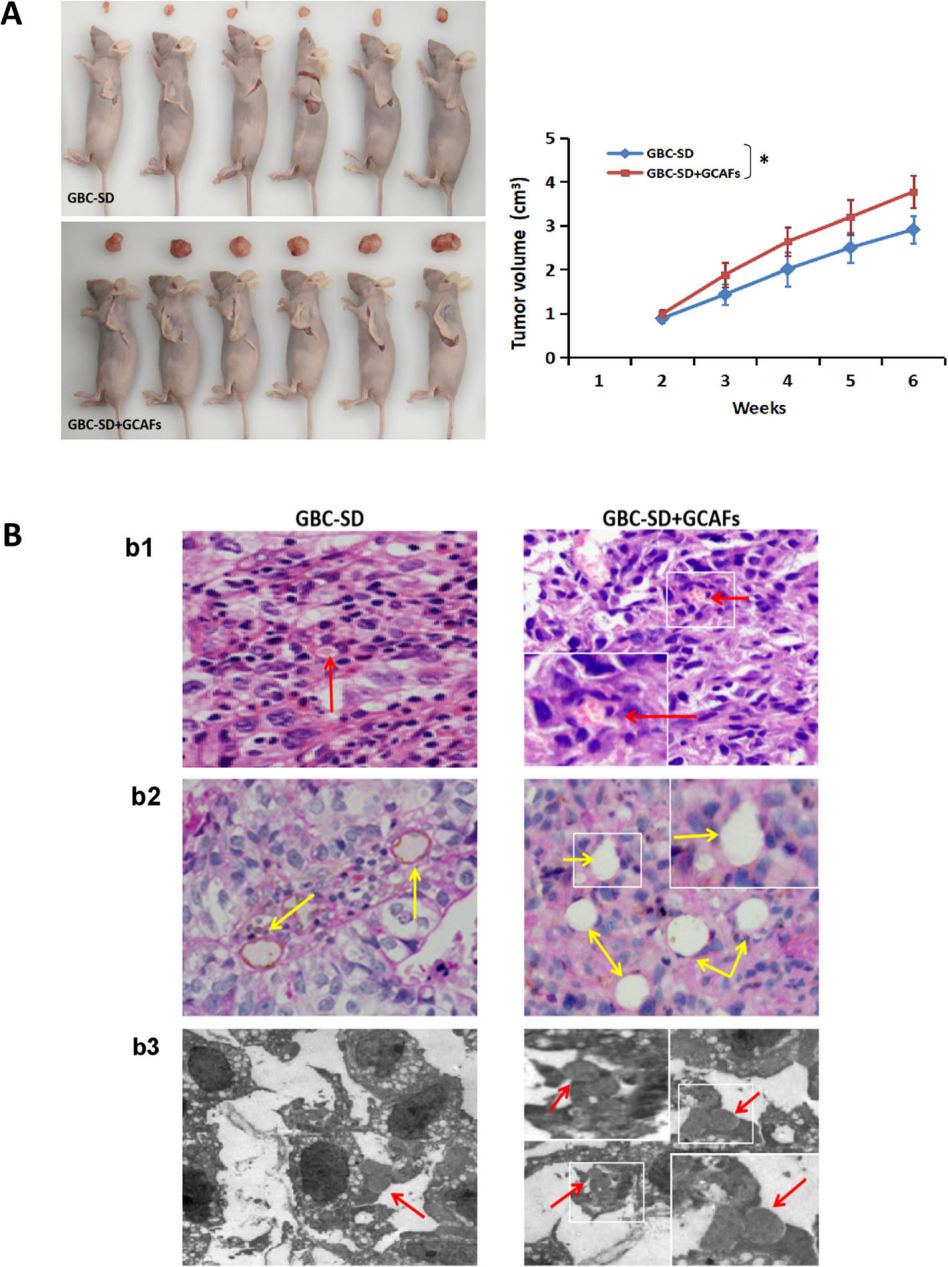
GCAFs promote the tumor growth and VM formation of GBC in vivo. **A.** Size, volume and growth curves of tumor xenografts in GBC-SD + GCAFs group were significantly larger than that of GBC-SD group (all *P < 0.05). **B.** (**b1.** HE staining, **b2.** CD_31_-PAS double staining; × 200). Sections of the xenografts in GBC-SD group and GBC-SD + GCAFs group showed tumor cell-lined vessel-like structure with single or multiple red blood cells inside (*red arrowhead*) without any evidence of tumor necrosis, and PAS-positive substances line the channel-like structures (*yellow arrowhead*). Of them, more tumor cell-lined vessel-like structures with multiple red blood cell inside and more PAS-positive substances lining the channel-like structures of the xenograft sections in GBC-SD + GCAFs group were observed. TEM (**B3.**; original magnification, × 8000) clearly visualized single (GBC-SD group) or multiple (GBC-SD + GCAFs group) red blood cells in the central of tumor nests in the xenografts (*red arrowhead*)

A nude mouse xenograft model was established to further verify whether GCAFs can promote VM formation and tumor growth of GBC in vivo. As shown in Fig. 3, size, volume and growth curves of tumor xenografts in GBC-SD + GCAFs groups were significantly larger than that of GBC-SD group (*P* < 0.05; Fig. 3A); H&E staining (Fig. 3Bb1) and CD_31_-PAS double staining (Fig. 3Bb2) of xenografts section in GBC-SD and GBC-SD + GCAFs groups showed that tumor cells were lined with vessel-like structure, with single or multiple red blood cell inside, without any evidence of tumor necrosis, and PAS positive substances were arranged in a channel-like arrangement; furthermore, TEM (Fig. 3Bb3) clearly visualized single or multiple red blood cells in the central of tumor nests in the xenografts; but the number of blood vessel-like structures, erythrocytes and PAS positive substances in GBC-SD + GCAFs group were significantly more than those of GBC-SD group (Fig. 3Bb1–2). These results further confirmed that GCAFs have the ability to promote tumor growth and VM formation in GBC.

### NOX4 is highly expressed in the process of VM formation triggered by GCAFs

**Fig. 4:**
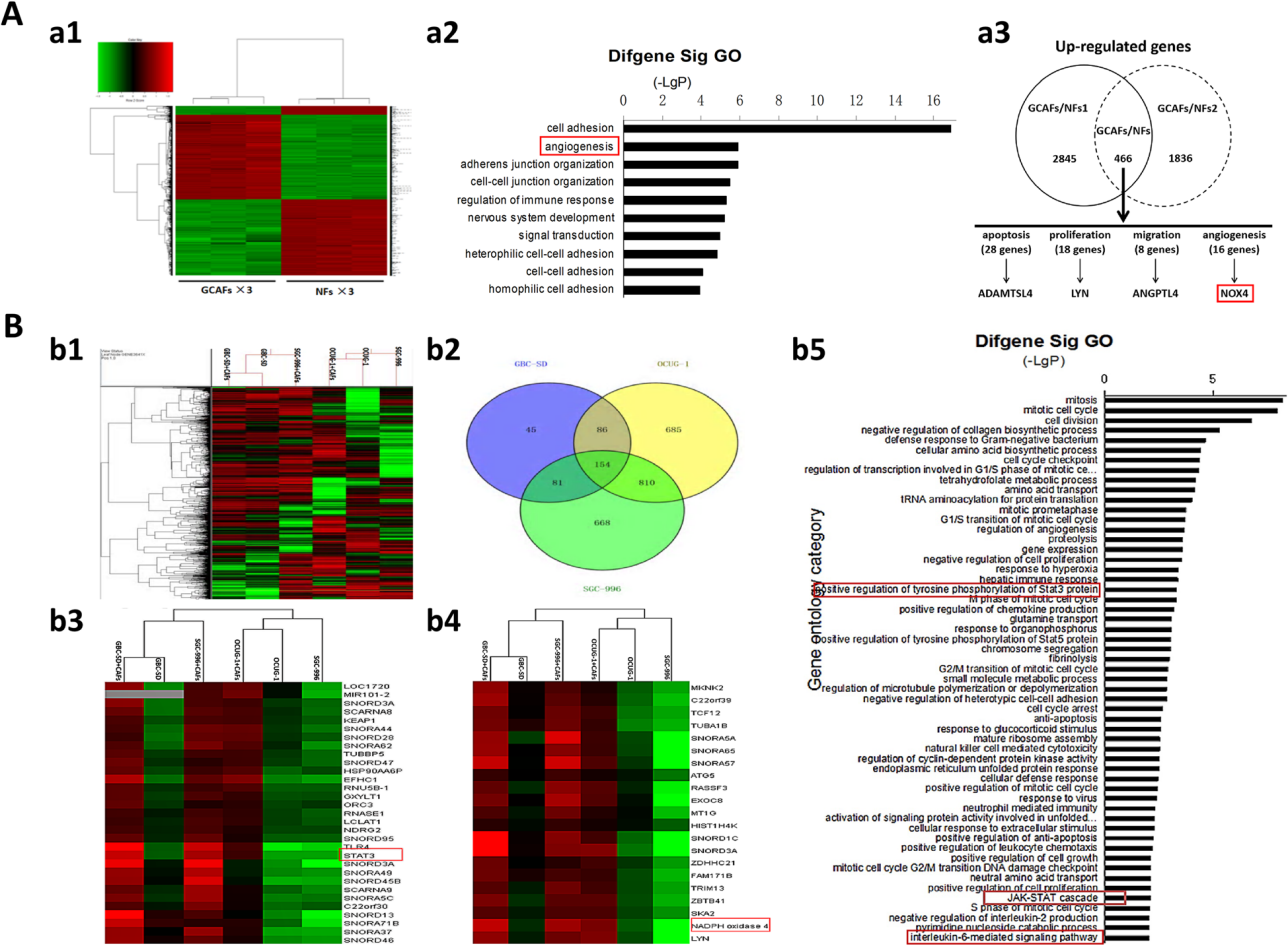
Microarray analysis of differential gene profiles in vivo. **A,** Affymetrix GeneChip Human 1.0ST array. (**a1**) Gene expression profile for GCAFs and NFs (red color denotes upregulation and green denotes downregulation). (**a2**) Gene ontology (GO) analysis (biological process) based on the classification of gene numbers, expression and significance probability. The red mark showed that the process of angiogenesis was significantly expressed. (**a3**) 466 upregulated genes (FC > 1.5) were identified in GCAFs/NFs, and NOX4 expression was significantly upregulated (FC = 2.58) in GCAFs compared with NFs. **B,** lncRNAs chip analysis of VM-related gene expression profile in GBC. (**b1**) Heatmap of gene chip. (**b2**) Venn modal analysis. (**b3-b4**) Venn modal analysis of upregulation genes showed the high-expression of STAT3 and NOX4 in 154 of VM-related genes. (**b5**), GO analysis, (upregulation, > 2 times). The results showed that the activation of IL-6 mediated JAK-STAT pathway during VM formation in GBC

In order to understand which genes are involved in the VM formation triggered by GCAFs in GBC, we analyzed the gene difference expression profile of GCAFs/NFs and VM(+)/VM(−) in vitro using Affymetrix GeneChip Human 1.0ST array and Affymetrix Human lncRNA array. As shown in Fig. 4A, a total of 466 upregulated genes (FC > 1.5) and 596 downregulated genes (FC < 0.67) were identified in GCAFs/NFs according to the inclusion criteria. As one of 16 angiogenesis-related genes, NOX4 was significantly upregulated (FC = 2.58) in GCAFs compared with NFs. Furthermore, Affymetrix Human lncRNA array was used to analyze VM-related gene expression profile in GBC. Venn modal analysis (upregulation, > 2 times; or downregulation, < 0.5 times) showed that the expression of NOX4, JAK and STAT3 was significantly upregulated in 154 VM-related genes (Fig. 4B). These data preliminary showed that NOX4, as a GCAFs-derived VM-related gene, is highly expressed in the progress of VM formation triggered by GCAFs in GBC.

**Fig. 5:**
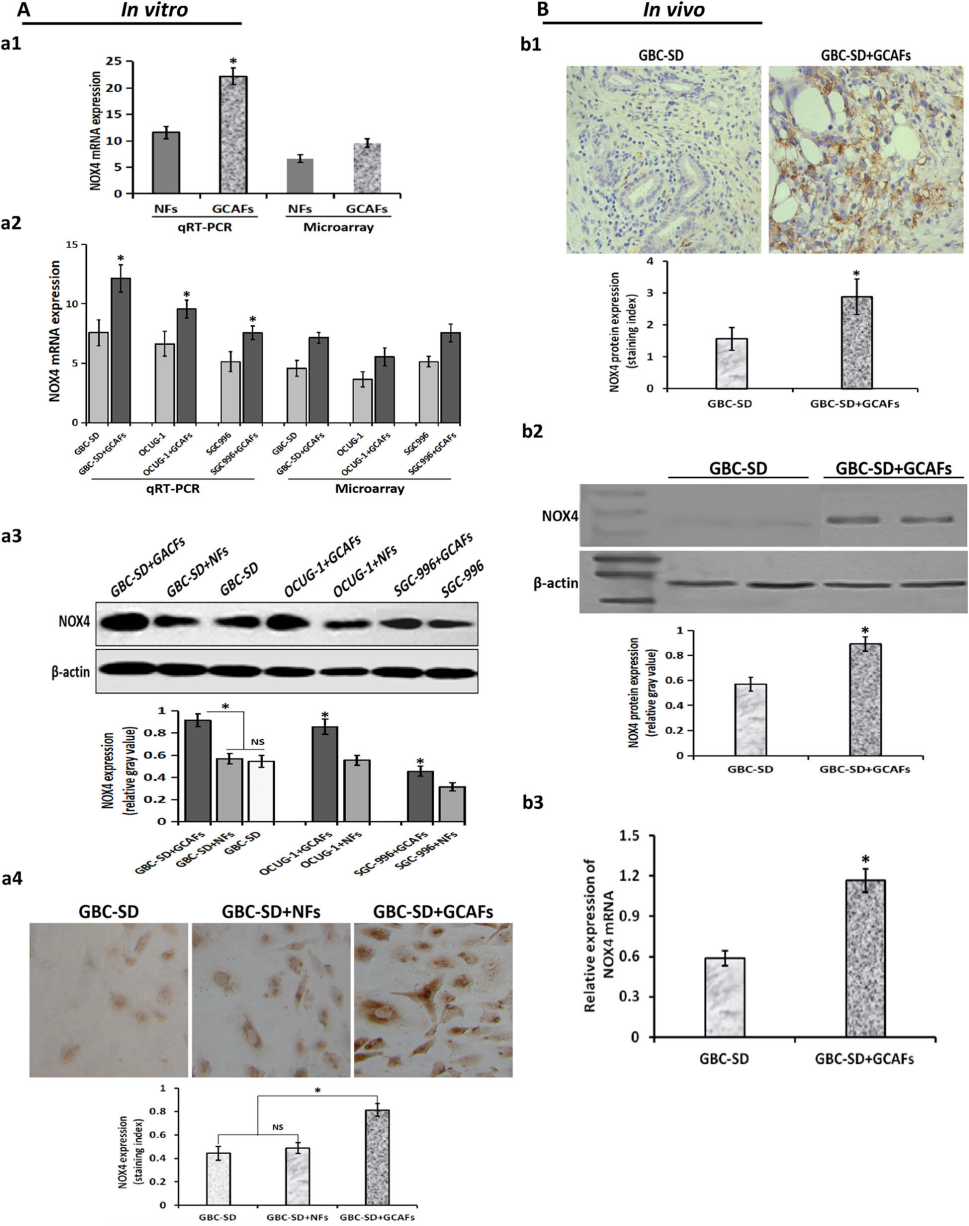
Verification of NOX4 expression during GCAFs-trggered VM formation in vitro and in vivo. **A**, in vitro. (**a1**) qRT-PCR in GCAFs/NFs. NOX4 mRNA expression in GCAFs was significantly upregulated compared to NFs (*P < 0.05). (**a2**) qRT-PCR in 3-D matrices. NOX4 mRNA expression in 3-D matrices of GBC cells (GBS-SD, SGC-996, OCUG-1) + GCAFs co-culture was significantly higher than that of alone GBC cells culture (all *P < 0.05). (**a3**) Western blotting in 3-D matrices. The expression of NOX4 protein in 3-D matrices of GBC cells+GCAFs co-culture was significantly upregulated compared with GBC cell+NFs co-culture and alone GBC cells culture (all *P < 0.05). (**a4**) IHC (× 200) in 3-D matrices. The expression of NOX4 protein in 3-D matrices of GBC-SD + GCAFs co-culture was significantly higher than that of GBC-SD + NFs co-culture or alone GBC-SD culture (all *P < 0.05), but no statistical difference was observed between GBC-SD + NFs group and GBC-SD group (*P* > 0.05). **B**, in vivo. (**b1**) IHC (× 200); (**b2**) western blotting. The expression of NOX4 protein in GBC-SD + GCAFs xenografts was significantly higher than that of GBC-SD xenografts (all *P < 0.05). (**b3**) qRT-PCR. The expression NOX4 mRNA in GBC-SD + GCAFs xenografts was significantly upregulated compared with GBC-SD xenografts (*P < 0.05)

To verify that NOX4 expression is upregulated during the formation of VM induced by GCAFs in GBC, we further detected the expression of NOX4 in vitro and in vivo using qRT-PCR, western blot and IHC. As shown in Fig. 5, NOX4 mRNA expression in GCAFs (Fig. 5A.a1) or in 3-D matrices of GBC cell (GBS-SD, SGC-996, OCUG-1) + GCAFs co-culture (Fig. 5A.a2) was significantly upregulated compared with NFs or alone GBC cells in vitro, which was consistent with the results of microarray analysis. And, the expression of NOX4 protein in GBC cell+GCAFs co-culture groups was significantly higher than that of GBC-SD + NFs co-culture or alone GBC-SD in vitro (all *P* < 0.05; Fig. 5A.a3–4), but no statistical difference was observed between GBC-SD + NFs and GBC-SD groups. Furthermore, the expression of NOX4 in GBC xenografts in vivo was detected by IHC (Fig. 5B.b1) and western blotting (Fig. 5B.b2). The results showed that NOX4 was also high expressed in GBC-SD + GCAFs group compared with GBC-SD group (all *P* < 0.05); and the expression of NOX4 mRNA in GBC-SD + GCAFs group was significantly upregulated compared with GBC-SD group (*P* < 0.05; Fig. 5B.b3). All together, these data verified that NOX4, as a GCAFs-derived VM-related gene, was highly expressed in VM formation triggered by GCAFs; in other words, GCAFs upregulated NOX4 expression in the process of VM formation in GBC.

**Fig. 6:**
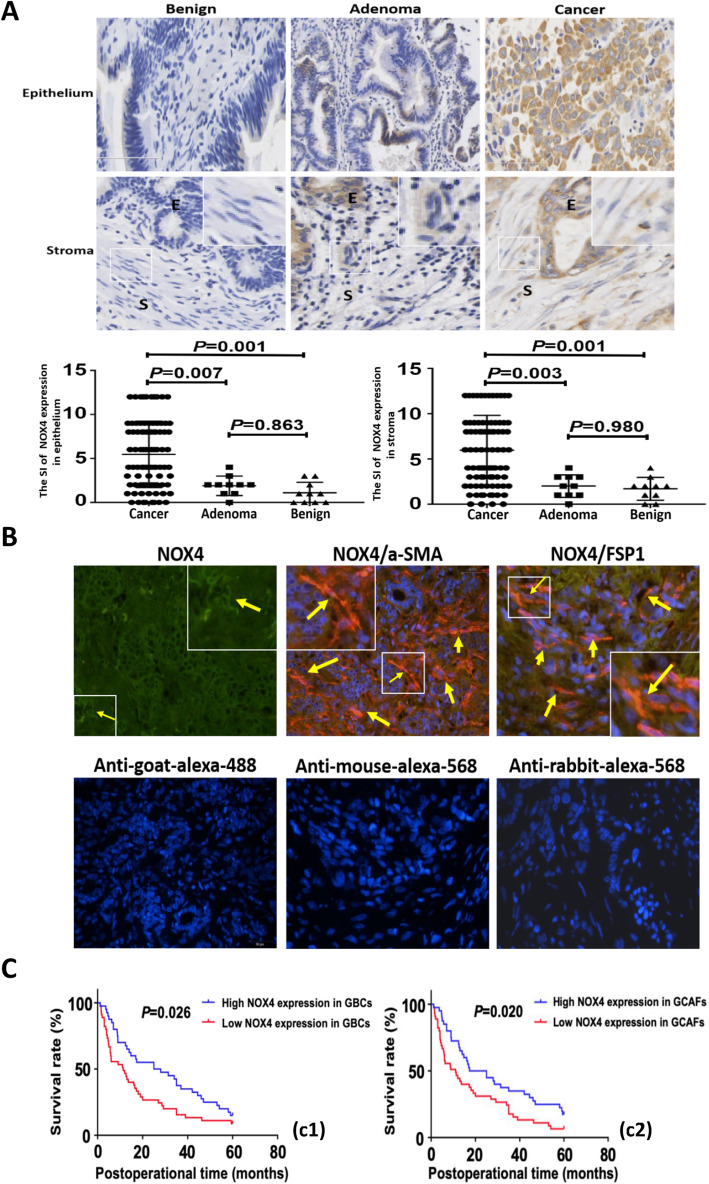
NOX4 is highly expressed in human GBC tissue/stroma, and is associated with poor prognosis. **A** (IHC, × 200; E = epithelium, S=Stroma)**.** The expression (cytoplasmic and/or nuclear brown staining) of NOX4 in epithelium or stroma of GBC (*n* = 85) was significantly upregulated compared with gallbladder precancerous (adenomas, *n* = 10; epithelium: 5.471 ± 0.410 vs. 1.900 ± 0.348, *P* = 0.007, stroma: 5.965 ± 0.419 vs. 2.000 ± 0.394, *P* = 0.003) and benign lesions (cholecystitis, n = 10; epithelium: 5.471 ± 0.410 vs. 1.100 ± 0.379; stroma: 5.965 ± 0.419 vs. 1.700 ± 0.396; both *P* = 0.001), but no statistical difference between precancerous lesions and benign lesions (both *P* > 0.05). Magnified insets show representative NOX4 staining in stroma. **B** (CIF staining, × 200)**.** The expression of NOX4 (green) co-localizes with α-SMA and FSP1 (red) positive stroma. Representative samples of NOX4, α-SMA and FSP1 are shown. Sections were counterstained with DAPI. Secondary antibody only controls are shown: anti-goat-alexa-488 for NOX4, anti-mouse-alexa-568 for α-SMA and anti-rabbit-alexa-568 for FSP1. Arrows and inset point to positive staining in fibroblastic cells. **C.** Kaplan-Meier analysis of GBC patients with high and low NOX4 expression in GBC cells/stroma. The survival time of GBC patients with high NOX4 expression in tumor cells or stroma was significantly shorter than that of GBC patients with low NOX4 expression (log-rank test, **c1**, GBCs; *P* = 0.026; **c2**, GCAFs, *P* = 0.020)

To further verify whether NOX4 was overexpressed in VM formation of GBC triggered by GCAFs, we detected the expression of NOX4 protein in human gallbladder tissue or stroma samples using IHC and CIF staining. As shown in Fig. 6, the staining index (SI) of NOX4 expression in GBC epithelium or stroma was significantly higher than that in gallbladder precancerous lesions (*P* = 0.007; *P* = 0.003) and benign lesions (both *P* = 0.001), but there was no significant difference in the expression of NOX4 between precancerous and benign lesions (*P* = 0.863) or stroma (*P* = 0.980) (Fig. 6A). In addition, CIF staining with stromal markers α-SMA and FSP-1 showed that NOX4 (green) overlapped or co-localized with α-SMA and FSP1 (red or brown) positive stroma in GBC (Fig. 6B). These results further confirmed that NOX4, as a key gene for VM formation, was over-expressed in GBCs-induced VM formation of GBC.

### High NOX4 expression is related to aggression and unfavorable prognosis of GBC patients

Here, we further studied the relationship between NOX4 expression and clinical, pathological and prognostic factors in GBC patients. SI score was used to distinguish NOX4 expression: IS ≤3, NOX4 low expression; > 3, high expression. In the tumor cells of GBC, NOX4 was highly expressed in 50 cases (58.8%) and low in 35 cases (41.2%); in stromal cells, the expression of NOX4 was high in 55 cases (64.7%) and low in 30 cases (35.3%). As shown in Table 1-2, chi-square analysis and spearman rank correlation analysis indicated that the expression of NOX4 in tumor/stromal cells was not related to age, gender, tumor location, tumor size, histological type, lymph node metastasis and resection method (all *P* > 0.05), but significantly associated with tumor differentiation, liver metastasis, vascular invasion, Nevin staging and VM (all *P* < 0.05) in GBC patients.

**Table 2:** Correlation between NOX4 expression in tumor stroma and clinicopathological parameters in patients with gallbladder cancer

Variable	NOX4 expression [n (%)]		***x***^**2**^ value (***P***)	Spearman rank correlation, r (***P***)
	Low	High		
**Age** (y)				
> 65	11 (34.3)	21 (65.7)	0.014 (0.885)	0.010 (0.886)
≤ 65	19 (35.8)	34 (64.2)		
**Gender**				
Male	18 (33.3)	27 (66.7) 28 (70)	0.006 (0.958)	0.009 (0.960)
Female	12 (30)			
**Tumor location**				
Bottom	14 (30.4)	32 (69.6)	1.651 (0.350)	0.055 (0.605)
Neck and other	16 (41)	23 (59)		
**Tumor size** (**cm**)				
> 3	20 (41.3)	26 (58.7)	2.889 (0.077)	0.325 (0.434)
≤ 3	10 (25.6)	29 (74.4)		
**Histological types**				
Adenocarcinoma	29 (37)	52 (63)	0.024 (0.922)	0.027 (0.755)
Other^a^	1 (33.3)	3 (66.7)		
**Differentiation degree**				
High	10 (68.7)	6 (31.3)	9.725 (0.009) ^b^	0.518 (0.001) ^b^
Moderate	12 (44.4)	15 (55.6)		
Poor	8 (19)	34 (81)		
**Liver metastases**				
(+)	9 (22.5)	31 (77.5)	5.691 (0.022) ^b^	0.324 (0.028) ^b^
(−)	21 (48.8)	24 (51.2)		
**Vascular invasion**				
(+)	17 (25.3)	50 (74.7)	8.678 (0.005) ^b^	0.462 (0.002) ^b^
(−)	13 (55.5)	5 (44.5)		
**Lymph node metastasis**				
(+)	18 (38.2)	29 (61.8)	0.021 (0.930)	0.026 (0.735)
(−)	12 (36.8)	26 (63.2)		
**Nevin staging**				
III, IV, V	19 (28.3)	48 (71.7)	4.433 (0.038) ^b^	0.322 (0.035) ^b^
I, II	11 (55.6)	7 (44.4)		
**Resection method**				
R1, R2	13 (31.7)	28 (68.3)	0.319 (0.572)	0.025 (0.768)
R0	17 (40.9)	27 (59.1)		
**VM**				
(+)	15 (68.2)	7 (31.8)	6.113 (0.020) ^b^	0.389 (0.021) ^b^
(−)	15 (23.8)	48 (76.2)		

^a^squamous cell carcinoma, adenosquamous carcinoma; ^b^*P* < 0.05: statistically significant

Furthermore, the Cox proportional hazard model was used to determine the influencing factors related to survival prognosis, i.e. OS rate of GBC patients (Table 3). The univariate analysis suggested that tumor histological type, differentiation degree, Nevin stage, liver metastasis, vascular invasion, lymph node metastasis, resection method, NOX4 expression in tumor cells/GCAFs were significant prognostic indicators for the OS of GBC patients (all *P* < 0.05). Therefore, these indicators were selected as parameters to be included in the same Cox regression model. Further multivariate analysis confirmed that differentiation degree, liver metastasis, vascular invasion and NOX4 stroma expression (all *P* < 0.05) were the independent prognostic factors for the OS of GBC patients.

**Table 3:** Cox model analysis of influencing factors of survival prognosis in patients with gallbladder cancer

Variable	Single factor			Multiple factors		
	HR	95% CI	***P***	HR	95% CI	***P***
**Gender**						
Male vs. female	0.972	0.609–1.554	0.907			
**Age** (y)						
> 65 vs. ≤65	0.808	0.517–1.262	0.348			
**Tumor site**						
Bottom vs. neck etc.	0.755	0.481–1.184	0.221			
**Tumor size** (cm)						
> 3.0 vs. ≤3.0	1.434	0.909–2.262	0.121			
**Histological types**						
Adenocarcinoma vs. others ^b^	0.255	0.106–0.613	0.002^*a*^			
**Differentiation degree**						
High vs. medium/low	0.243	0.114–0.514	0.000^*a*^	0.437	0.196–0.975	0.039 ^*a*^
**Nevin staging**						
III ~ V vs. I ~ II	2.482	1.227–5.021	0.011^*a*^			
**Liver invasion**						
(+) vs. (−)	2.075	1.294–3.327	0.002^*a*^	1.949	1.187–3.201	0.012 ^*a*^
**Vascular invasion**						
(+) vs. (−)	3.235	1.911–5.475	0.000^*a*^	2.569	1.486–4.444	0.003 ^*a*^
**Lymph node metastasis**						
(+) vs. (−)	1.708	1.057–2.762	0.029^*a*^			
**Resection method**						
R1, R2 vs. R0	0.413	1.672–5.041	0.000^*a*^			
**NOX4 expression in cancer cells**						
High vs. low	1.985	1.484–3.592	0.005^*a*^			
**NOX 4 expression in stroma cells**						
High vs. low	1.628	1.389–3.329	0.035^*a*^	1.767	1.062–3.942	0.022 ^*a*^

^a^*P* < 0.05, statistically significant; ^b^adenocarcinoma, squamous cell carcinoma, adenosquamous carcinoma; HR, Hazard ratio; CI, confidence interval

Finally, Kaplan-Meier analysis and the log-rank test were used to further evaluate the effect of NOX4 expression on the survival of GBC patients. The mean, median and a 5-year survival rate for survival time of the high NOX4 expression in tumor cells (50/85, 58.8%) were 15.6 months 5.8 months and 10.5%, compared with 30.5 months 25.0 months and 22.2% for the low NOX4 expression (35/85, 41.2%). The mean, median and a 5-year survival rate for survival time of the high NOX4 expression in GBC stroma (55/85, 64.7%) were 15.7 months 4.8 months and 10.3%, compared with 26.7 months 16.1 months and 19.4% for the low NOX4 expression (30/85, 35.3%). As shown in Fig. 6C, Kaplan-Meier analysis showed that the survival time of GBC patients with high NOX4 expression in GBC tumor (Fig. 6C1, *P* = 0.026) and stroma (Fig. 6C2, *P* = 0.020) was significantly shorter than that of GBC with low NOX4 expression.

All together, these results confirmed that the expression of NOX4, especially in the tumor stroma, is an independent prognostic factor of GBC. The high expression of NOX4 in GBC tumor/stroma is closely related to VM formation, and indicates poor prognosis.

### GCAFs promote VM formation and tumor growth in GBC via upregulating NOX4 expression through IL-6/JAK/STAT3 signaling pathway

**Fig. 7:**
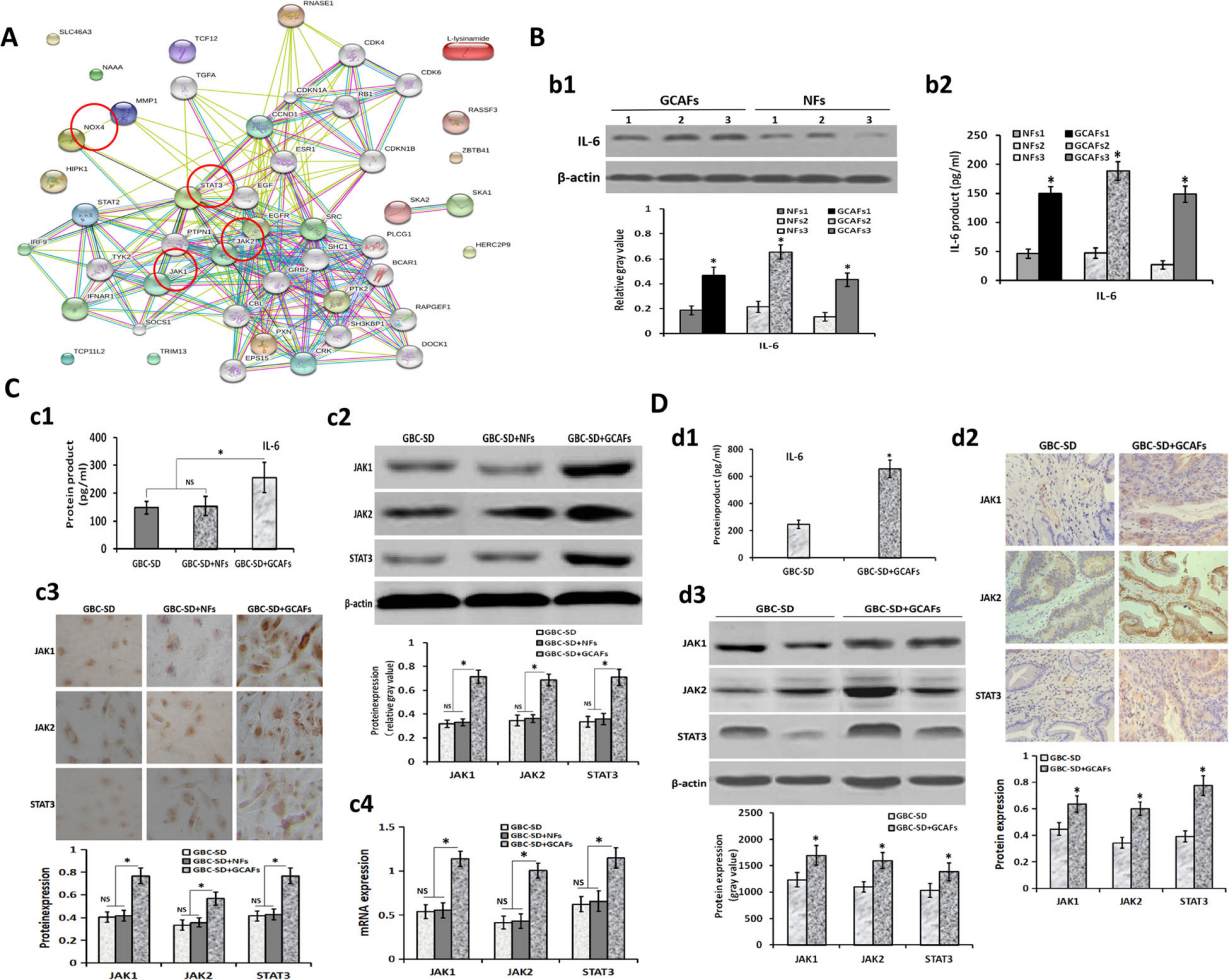
Verification of the pathway genes based on microarray analysis. **A,** A network regulation model of GCAFs promoting VM formation of GBC. NOX4, STAT3, JAK1 and JAK2 are regulated in the VM-related key genes. **B**, Confirmation of IL-6 expression in GCAFs/NFs in vitro (**b1**, Western blotting; **b2**, ELISA). IL-6 protein and product were highly secreted and upexpressed in GCAFs compared with NFs (all *P < 0.05). **C-D,** Verification the expression of IL-6/JAK/STAT3 pathway genes in vitro and in vivo. **C** (in vitro): **c1** (ELISA), the expression of IL-6 product in the supernatant of GBC-SD + GCAFs co-culture was significantly higher than that of GBC-SD or GBC-SD + NFs co-culture (*P < 0.05); **c2-c3** (**c2**, IHC, × 200; **c3**, western blotting), the expression of JAK1, JAK2 and STAT3 proteins in the 3-D co-culture matrices of GBC-SD + GCAFs was significantly higher than that of GBC-SD or GBC-SD + NFs co-culture (all *P < 0.05); **c4** (qRT-PCR), the expression of JAK1, JAK2 and STAT3 mRNAs in the 3-D co-culture matrices of GBC-SD + GCAFs was significantly upregulated compared with GBC-SD or GBC-SD + NFs co-culture (all *P < 0.05). But, no statistical difference was observed in the expression of JAK1, JAK2 and STAT3 at protein or mRNA level between GBC-SD and GBC-SD + NFs groups. **D** (in vivo): **d1** (ELISA), the expression of IL-6 product in the supernatant of GBC-SD + GCAFs group was significantly upregulated when compared with GBC-SD in xenografts (*P < 0.05); **d2-d3** (**d2**, IHC, × 200; **d3**, western blotting), The expression of JAK1, JAK2 and STAT3 proteins in GBC-SD + GCAFs group was significantly higher than that of GBC-SD group in xenografts (*P < 0.05)

In order to explore the underlying molecular mechanism of GCAFs promoting VM formation and tumor growth in GBC, we further analyzed the results of lncRNA microarray. As shown in Fig. 4B, Venn modal analysis showed that the expression of STAT3 and NOX4 was significantly increased among the 154 VM-related genes in GBC. Meanwhile, GO analysis showed that the activity of IL-6-mediated JAK-STAT pathway was significantly increased in the process of VM formation in GBC. So we constructed a network regulation model of GCAFs promoting VM formation and tumor growth in GBC. As shown in Fig. 7A in this network regulation model, NOX4, STAT3 and JAK were regulated in the VM-related key genes. These results suggested that GCAFs promote VM formation by upregulating NOX4 expression, and the mechanism may be related to the paracrine IL-6-mediated IL-6/JAK/STAT3 signal pathway.

In order to verify the hypothesis, we first detected the expression of IL-6 in GCAFs/NFs in vitro using western blotting and ELISA. As shown in Fig. 7B, IL-6 and its product were highly secreted and overexpressed in the culture supernatant of GCAFs compared with NFs in vitro (all *P* < 0.05). Furthermore, we detected the expression of related signaling pathway genes in 3-D culture/co-culture matrices using ELISA, western blotting and qRT-PCR. The results showed that the expression of IL-6 product in the supernatant of 3-D co-culture matrices, and the expression of JAK1, JAK2 and STAT3 at protein and mRNA levels in the 3-D co-culture matrices of GBC-SD + GCAFs was significantly higher than that of GBC-SD culture or GBC-SD + NFs co-culture (all *P* < 0.05); but no statistical difference was observed in the expression of these genes between GBC-SD and GBC-SD + NFs groups (Fig. 7C). Finally, we detected the expression of related signaling pathway genes in the nude mouse xenografts in vivo using ELISA, IHC and western blotting (Fig. 7D). The results showed that the expression of IL-6 in the supernatant of GBC-SD + GCAFs group was significantly higher than that of GBC-SD group (*P* < 0.05), and the protein expression of JAK1, JAK2 and STAT3 in GBC-SD + GCAFs group was significantly higher than that in GBC-SD group (all *P* < 0.05). All together, these results suggested that GCAFs promote VM formation and tumor growth in GBC via upregulating NOX4 expression through paracrine IL-6 mediating IL-6/JAK/STAT3 signaling pathway.

## Discussion

The interaction between TME and tumor cells has been a hotspot in the study of carcinogenesis [4, 5]. CAFs, as the most critical stromal cells in TME, participate in the process of tumor growth and development [6–13]. As an important supplement to tumor microcirculation, VM occurs in certain highly aggressive malignancies, and is closely related to tumor progression and poor prognosis [18–21]. However, the relationship between CAFs and VM formation has rarely been reported. Recently, some studies have confirmed that CAFs can promote VM formation in some tumors, such as HCC [30] and gastric cancer [31, 32]. In this study, we observed that GCAFs can promote VM formation and tumor growth of GBC in vitro and in vivo. Therefore, we first believed that GCAFs has the ability to promote tumor growth and VM formation in GBC.

Recently, it was reported that CAFs promote VM formation in HCC cells by secreting TGF-β and SDF1 [30]; CAFs-derived HGF promote vascularization in gastric cancer [31]. So whether there are specific genes regulation in the process of GCAFs-triggered VM formation in GBC? Here, we performed microarray analysis for GCAFs/NFs and VM (+)/VM (−). The results showed that NOX4 was the key gene for VM formation in GBC. Then, we detected NOX4 expression in vitro and in vivo, and confirmed that NOX4 was not only highly expressed in GCAFs, but also in human GBC tissue and stroma. As mentioned above, NOX4, as a member of the NADPH oxidase family, plays vital roles in proliferation, metastasis and angiogenesis of tumors through the production of ROS [40, 42, 44]. It has been confirmed that NOX4 is highly expressed in many types of tumor cells such as pancreatic cancer [45], renal cell carcinoma [46] and gastric cancer [47]. However, current researches on NOX4 are mainly focused on tumor cells, and the role of NOX4 in VM formation is rarely mentioned. Thus, our finding firstly confirmed that NOX4 is highly expressed in GBC and its stroma, which is a GCAFs-derived key gene for VM formation.

In order to investigate the clinical value of NOX4 overexpression, we further studied the relationship between NOX4 expression and clinicopathological characteristics/prognostic factors in GBC patients. It was recently reported that the expression of NOX4 is closely related to the tumor size and prognosis in gastric cancer [47]; the prognosis of colorectal cancer patients with high expression of NOX4 was poor [48]. In this study, we found that the high expression of NOX4 in GBC, especially in its stroma, was significantly correlated with tumor differentiation, liver metastasis, vascular invasion and Nevin staging, especially VM formation; and the expression of NOX4 in stroma was one of the independent prognostic factors for the OS of GBC patients. The survival time of GBC patients with high NOX4 expression was significantly shorter than that of GBC patients with low expression. So we firstly confirmed that high NOX4 expression is related with aggression and unfavorable prognosis in patients with GBC. This finding may contribute to the establishment of novel biomarkers and provide help to evaluate the progression and prognosis of GBC patients.

At present, the mechanism of CAFs promoting tumor VM formation is not clear. It was reported that CAFs promoted VM formation in HCC by paracrine TGF-β and SDF1 [30]; CAFs-derived HGF promoted vascularization in gastric cancer via PI3K/AKT and ERK1/2 signaling [31]; CAFs induced VM formation in gastric cancer cells through EphA2-PI3K pathway [32]. In view of aforementioned microarray analysis results, we found that the expression of IL-6-mediated JAK-STAT pathway genes was significantly upregulated. Meanwhile, among 154 VM-related genes, NOX4, JAK and STAT3 were involved in the formation of VM in GBC. So, we constructed a gene network regulation model in which GCAFs promote VM formation, and found that JAK1, JAK2, STAT3 and NOX4 were regulated in the VM-related key genes.

Paracrine is one of the important ways in which CAFs act on tumor cells, CAFs can promote tumor cell growth and angiogenesis by secreting a variety of cytokines [49, 50]. IL-6 is an important inflammatory cytokine secreted by tumor cells or stromal cells [51], which is highly expressed in some tumors, and participate in a variety of malignant biological processes by activating a variety of signal pathways, such as STAT3, ERK and MAPK [52–55]. It is reported that IL-6-mediated JAK/STAT3 pathway plays an important role in tumor proliferation, invasion and metastasis [56]. So, IL-6/JAK/STAT3 signaling is believed as a key signaling pathway for tumor progression, and has been confirmed in many types of solid tumors such as HCC [57], breast cancer [58] and lung adenocarcinomas [59]. According to the results of lncRNA analysis, we considered that the process of GCAFs promoting VM formation in GBC may be mediated by IL-6/JAK/STAT3 pathway. In order to verify the hypothesis, we detected the mRNA and protein expression of IL-6, JAK1, JAK2 and STAT3 in vitro and in vivo. The results confirmed that IL-6 was highly secreted and expressed in GCAFs, and these pathway genes were highly expressed in the process of VM formation induced by GCAFs.. All together, these results firstly suggested that GCAFs promote VM formation and tumor growth in GBC via upregulating NOX4 expression through paracrine IL-6 mediating IL-6/JAK/STAT3 signaling pathway. Therefore, this study may provide a new theoretical basis for exploring the mechanism of VM formation in GBC, and provide a potential therapeutic target for anti-VM therapy.

## Conclusions

Collectively, our study firstly demonstrate that GCAFs can upregulate NOX4 expression to promote VM formation and tumor growth in GBC, which may be achieved by paracrine IL-6-mediated IL-6-JAK-STAT3 signal pathway. NOX4, as a key gene for VM formation in GBC, is highly expressed in the tumor and stroma, which is related to the progression and poor prognosis of GBC patients. The present findings may provide a new idea and approach for the diagnosis, prognosis and anti-VM target treatment of human GBCs.

## Data Availability

The datasets and materials used during the current study are available from the corresponding author on reasonable request.

## Abbreviations

GBC: Gallbladder cancer
CAFs: Cancer-associated fibroblasts
GCAFs: Gallbladder cancer-associated fibroblasts
NFs: Normal fibroblasts
VM: Vasculogenic mimicry
TME: Tumor microenvironment
ECM: Extracellular matrix
NOX4: Nicotinamide adenine dinucleotide phosphate oxidase 4, NADPH oxidase 4
ROS: Reactive oxygen species
DMEM: Dulbecco’s modified Eadles medium
HUVEC: Human umbilical vein endothelial cell
α-SMA: α-smooth muscle actin
FAP: Fibroblast activation protein
IHC: Immunohistochemistry
CIF: Co-immunofluorescence
FBS: Fetal bovine serum
PBS: Phosphate buffer saline
CO_2_: Carbon dioxide
3-D: Three-dimensional
HE: Hematoxylin and eosin
PAS: Periodic acid-Schiff
DAB: 3,3-diaminobenzidine
OD: Optical density
SPF: Specific pathogen-free
TEM: Transmission electron microscopy
OS: Overall survival
IVT: In vitro transcription
FC: Fold change
GO: Gene Ontology
SI: Staining index
ELISA: Enzyme-linked immunosorbent assay
IL-6: Interleukin-6
DAPI: Diamidine phenylindole
qRT-PCR: Quantitative reverse transcription-polymerase chain reaction
JAK: Janus kinase
STAT: Signal transducer and activator of transcription
GAPDH: Glyceraldehyde-3-phosphate dehydrogenase
RIPA: Radioimmunoprecipitation assay
ECL: Enhanced chemiluminescent
HR: Hazard ratio
CI: Confidence interval
