# Correction to: Gallbladder cancer-associated fibroblasts promote vasculogenic mimicry formation and tumor growth in gallbladder cancer via upregulating the expression of NOX4, a poor prognosis factor, through IL-6-JAK-STAT3 signal pathway

**DOI:** 10.1186/s13046-021-02014-5

**Published:** 2021-07-17

**Authors:** Mu-Su Pan, Hui Wang, Kamar Hasan Ansari, Xin-Ping Li, Wei Sun, Yue-Zu Fan

**Affiliations:** 1 Department of Surgery, Tongji Hospital, Tongji University School of Medicine, Tongji University, Shanghai, 200065 P.R. China; 2 Department of Surgery, Shanghai Tenth People’s Hospital, Tongji University School of Medicine, Tongji University, Shanghai, 200072 P.R. China

**Correction to: J Exp Clin Cancer Res 39, 234 (2020)**

**https://doi.org/10.1186/s13046-020-01742-4**

Following publication of the original article [1], the authors identified some minor errors in image-typesetting in Fig. 2, specifically:

- Fig. 2a: GBC-SD +NFs panel (top row, middle) has been replaced with the correct image
- Fig. 2a: TJ-GBC2 +GCAFs panel (middle row, right) has been replaced with the correct image
- Fig. 2a: SGC-996 +NFs panel (bottom row, middle) has been replaced with the correct image
- Fig. 2b: SGC-996 +NFs panel (bottom row, middle) has been replaced with the correct image
- Fig. 2c: both TJ-GBC2 +NFs 8h and 24h panels (middle row, middle) have been replaced with the correct images
- Fig. 2d: +GBC-SD+NFs panel (top row, middle-right) has been replaced with the correct image

The corrected figure is given below. The correction does not have any effect on the results or conclusions of the paper. The original article has been corrected.

**Fig. 2:**
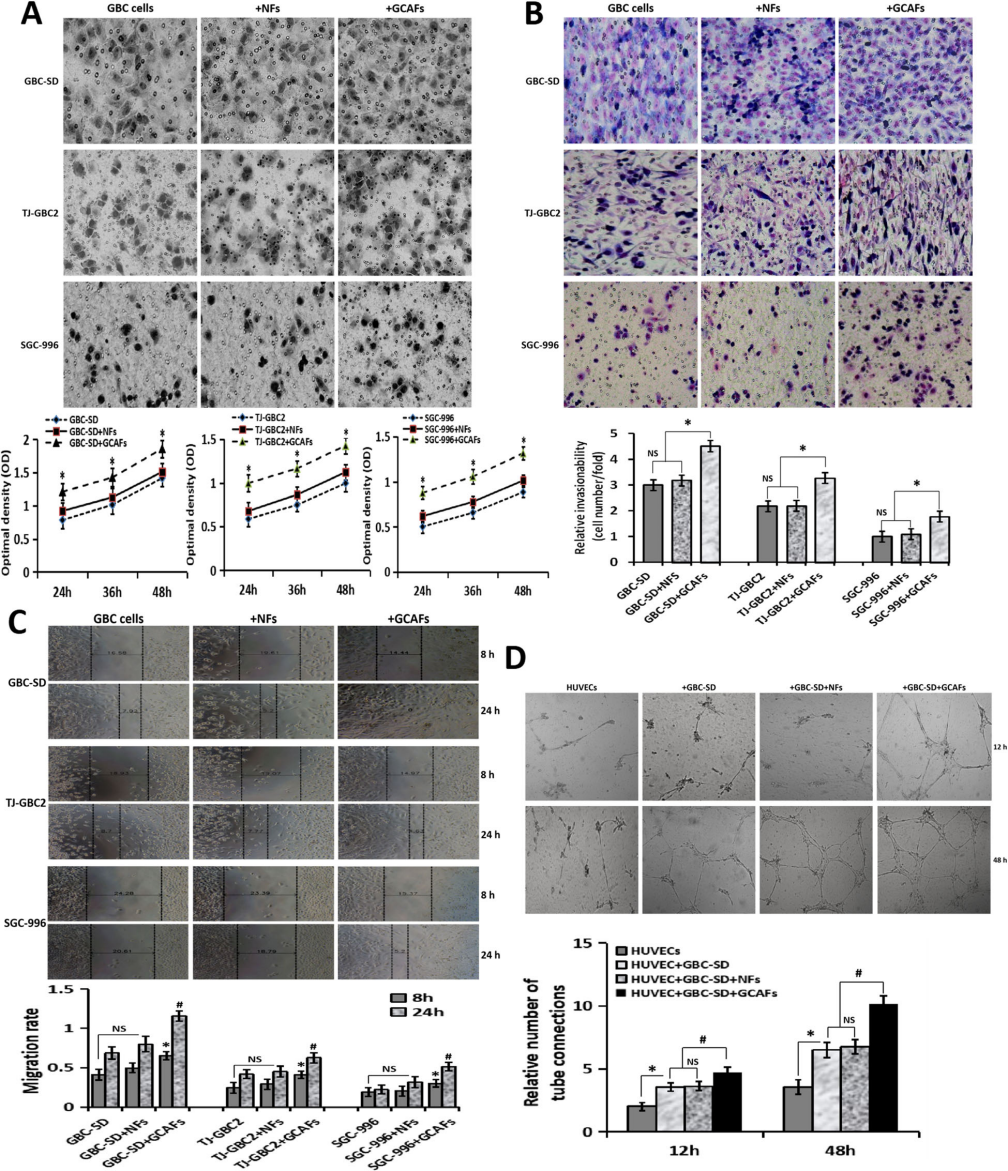
GCAFs promote the malignant phenotypes of GBC cells/HUVECs. **A**, Proliferation assay. Compared with NFs, GCAFs significantly promoted the proliferation of GBC cells at 24 h, 36 h and 48 h (vs. GBC cells group and GBC cells+NFs group, all *P < 0.05). **B**, Transwell invasion assay (Giemsa stain, × 200). The number (relative invasion ability) of cells that invaded through the basement membrane in GBC cell+GCAFs group was significantly more than that of GBC cell group and GBC cell+NFs group (all *P < 0.05). **C**, Wound healing assay. The cell migration rate of GBC cell+GCAFs group was significantly stronger than that of GBC cell group and GBC cell+NFs group at 8 h and 24 h (all ^#^*P* < 0.05). **D**, Tube formation assay. At 12 h: HUVEC group (−); HUVEC+GBC-SD and HUVEC+GBC-SD + NFs (+), (vs. HUVEC, all *P < 0.05); HUVEC+GBC-SD + GCAFs (+), (vs. HUVEC+ GBC-SD or HUVEC+GBC-SD + NFs, all ^#^*P* < 0.05). At 48 h: obvious tubular formation was observed in all the four groups, the number of tube formed in HUVEC+GBC-SD + GCAFs group was significantly more than that in the other three groups (all ^#^*P* < 0.01), but no statistical difference was observed between HUVEC+GBC-SD group and HUVEC+GBC-SD + NFs group (*P* > 0.05)
